# DNase I levels and disease outcome in JIA patients treated with etanercept

**DOI:** 10.1186/1546-0096-9-S1-P171

**Published:** 2011-09-14

**Authors:** D Lazarević, J Vojinović, G Sušić, N Damjanov, J Bašić

**Affiliations:** 1 Department of Pediatric Rheumatology, University Clinical Center Niš, Serbia; 2 Institute of Rheumatology, Belgrade, Serbia; 3 Department of Biochemistry, Faculty of Medicine, Niš, Serbia

## Background

Failure to efficiently degrade the DNA of apoptotic cells activates innate immunity, by induction of TNFά and IFNβ production, causing chronic arthritis. If deficient, DNase I could leed to accumulation of undigested DNA which induce activation of phagocytes and production of proinflammatory cytokines, notably TNF.

## Aim

Disease outcome in JIA patients after one year of treatment with TNFά therapy and their DNase I levels.

## Methods

The study was performed in 25 JIA patients who donated paired serum samples prior and one year after continous etanercept therapy. Basic clinical data (six core set variables defined in ACR PEDI outcome score) were recorded along with alkalyne DNase I serum levels using the method where acid soluble nucleotides are determined spectrophotometrically at 260 nm. Treatment schedule of etanercept was 0,4mg/kg body weight subcutaneously twice weekly.

## Results

JIA patients mean age was 14,7+/-4,22 and disease duration is 6,59+/- 2,76. Disease type distribution was 8% systemic, 28% polyarticular RF-, 25% polyarticular RF+, 17% ERA and 21% extended oligoarticular JIA. Summary of data results prior and after anti TNFά therapy: ESR 26,88 vs.15,52 (p<0,01); patientVAS 40,24 vs.24,40 (p<0,05); physicianVAS 38,08 vs.10,32 (p<0,01); CHAQ 0,674 vs.0,375 (p<0,01); LOM 15,52 vs. 11,68 (NS); AA 9,24 vs.2,64 (p<0,01). DNase I levels were significantly lower prior (2.934 U/l) compared to values after one year therapy (4,184 U/l; p<0,01). We have found correlation between DNase I levels and AA (r=-0,993 p<0,5) and other clinical outcome variables prior and after therapy.

## Conclusion

JIA patients with active disease have decreased DNase I levels. Our results indicate significant increase of DNase I in the sera of JIA patients after one year of anti TNFά therapy which was associated to the disease clinical improvement.

